# The demographic and clinical characteristics of a turkish enthesitis-related arthritis cohort: a single center experience

**DOI:** 10.1186/1546-0096-12-S1-P46

**Published:** 2014-09-17

**Authors:** Balahan Makay, Özge Altuğ Gücenmez, Deniz Bayraktar, Erbil Ünsal

**Affiliations:** 1 Pediatric Rheumatology, Dokuz Eylül University Hospital, İzmir, Turkey; 2 School of Physiotherapy and Rehabilitation, Dokuz Eylül University Hospital, İzmir, Turkey

## Introduction

Enthesitis-related arthritis is a subgroup of JIA, which is encountered more frequently in Turkish patients than the European populations. Our knowledge about the difference of disease charecteristics between Turkish ERA patients and other populations are limited.

## Objectives

To evaluate the demographic and clinical characteristics of a Turkish ERA cohort and to identify the distinguishing features of patients who required anti-TNF α treatment compared to the patients who did not.

## Methods

The hospital charts of patients who were diagnosed as ERA in our department between 2000-2013 according to the ILAR criterion were retrospectively evaluated. The demographic and clinical characteristics of patients were recorded. The characteristics of patients in whom anti-TNF α treatments were indicated were compared with the other patients on conventional therapies.

## Results

A total of 100 patients were included. Eighty five percent were males. The mean age at onset of disease was 11.7 ± 2.9 years, age at diagnosis was 13.2 ± 2.8 years and delay time for diagnosis was 1.5 ± 1.8 years. There was a negative correlation between the age at disease onset and delay time for diagnosis (r=-382, p=0.000). The family history for anyklosing spondylitis was present in %32 patient. HLA B27 was available in 87 patients and positive in 60 % of them. Erythrocyte sedimentation rate (ESR) was high in 69 patients and normal in 31 at the time of diagnosis. Enthesitis was positive in 65 patients, the most common site being the Achilles followed by plantar fascial insertion at calcaneus. Fifty patients had radiologically proven sacroiliitis (14 unilateral, 36 bilateral). Thirty six patient had hip involvement (19 unilateral, 17 bilateral). Fifty seven patients had ankle and/or knee involvement. Tarsitis was present in 22 patients. Only 7 patients had anterior uveitis. The treatments used were as follows: systemic corticosteroid (n=31), intraarticular steroid injection (n=20), methotrexate (n=28), sulphasalazine (n=97). Anti-TNF α treatments were used in 21 patients (etanercept 13, adalimumab 8). High ESR, tarsitis, sacroiliitis, hip involvement and systemic corticosteroid use were found associated with anti-TNF α requirement. There was not a significant association between the delay time for diagnosis and anti-TNF α use.

## Conclusion

The presented Turkish cohort displayed some remarkable differences from other series reported in the literature:

- Lower frequency of HLA B27 positivity

- Lower frequency of anterior uveitis

The patients who had the undermentioned characteristics at the time of diagnosis were seen to have more severe disease that required anti-TNF treatment:

- High ESR

- Tarsitis

- Sacroiliitis

- Hip involvement

- Systemic corticosteroid use

## Disclosure of interest

None declared.

